# Characterization and evaluation of 2.5 MV electronic portal imaging for accurate localization of intra‐ and extracranial stereotactic radiosurgery

**DOI:** 10.1120/jacmp.v17i4.6247

**Published:** 2016-07-08

**Authors:** Kwang Hyun Song, Karen Chin Snyder, Jinkoo Kim, Haisen Li, Wen Ning, Robert Rusnac, Paul Jackson, James Gordon, Salim M. Siddiqui, Indrin J. Chetty

**Affiliations:** ^1^ Radiation Oncology, Henry Ford Health System Detroit MI; ^2^ Texas Oncology Fort Worth TX USA

**Keywords:** 2.5 MV portal imaging, stereotactic radiosurgery, stereotactic body, radiation therapy

## Abstract

2.5 MV electronic portal imaging, available on Varian TrueBeam machines, was characterized using various phantoms in this study. Its low‐contrast detectability, spatial resolution, and contrast‐to‐noise ratio (CNR) were compared with those of conventional 6 MV and kV planar imaging. Scatter effect in large patient body was simulated by adding solid water slabs along the beam path. The 2.5 MV imaging mode was also evaluated using clinically acquired images from 24 patients for the sites of brain, head and neck, lung, and abdomen. With respect to 6 MV, the 2.5 MV achieved higher contrast and preserved sharpness on bony structures with only half of the imaging dose. The quality of 2.5 MV imaging was comparable to that of kV imaging when the lateral separation of patient was greater than 38 cm, while the kV image quality degraded rapidly as patient separation increased. Based on the results of patient images, 2.5 MV imaging was better for cranial and extracranial SRS than the 6 MV imaging.

PACS number(s): 87.57.C

## I. INTRODUCTION

Image‐guided radiation therapy (IGRT) utilizes one or more imaging techniques to improve target localization in treatment rooms. The tumor targeting accuracy is strongly dependent on the quality of images as well as the positioning hardware precision. With the emergence of electronic portal imaging devices, imaging frequency during radiation treatment has increased remarkably with the convenience of accessing and reviewing patient images instantly.^1^, ^2^ Higher imaging frequency consequently improved both patient positioning and delivery accuracy, leading to better tumor control with reduced normal tissue complication.^3^, ^4^, ^5^, ^6^ As the radiation planning and delivery techniques become more sophisticated, it is of great importance to have a high quality imaging and guidance system. Furthermore, it is a must to have a precision submillimeter accurate IGRT system for single‐fraction or hypofractionated stereotactic radiosurgery (SRS) or stereotactic body radiotherapy (SBRT).^7^, ^8^, ^9^, ^10^, ^11^, ^12^, ^13^

For the past 15 years, the electronic portal imaging devices (EPID) have improved significantly, producing high contrast and high resolution images.^14^, ^15^, ^16^, ^17^ However, the intrinsic contrast of the conventional 6 megavoltage (MV) portal imaging is limited due to the significantly higher amount of Compton interactions than photoelectric interactions in human tissue. In Compton interaction, an incoming X‐ray photon scatters before reaching the imager, degrading image quality, whereas, in photoelectric interaction, a photon is totally absorbed and a photoelectron is ejected as a result. This process produces shadow and eventually develops contrast in radiographs.^18^, ^19^ Therefore, image quality in X‐ray imaging can be enhanced by having more photoelectric interactions, which is predominant in the lower energy range. Various research groups have performed Monte Carlo investigations and experiments using low‐Z target materials in linacs to improve imaging performance^20^, ^21^, ^22^, ^23^ Recent studies have revealed that the relative fraction of photons between 25–150 keV in a 2.5 MV commercial imaging beam is 22%, whereas only 0.3% in 6 MV treatment beam.^21^, ^22^ The amount of photons in this diagnostic range can improve the imaging contrast in a phantom. Recently, a 2.5 MV portal imaging has been released on the Varian TrueBeam machine (Varian Medical Systems, Palo Alto, CA). However, its imaging capability and quality have not been evaluated. In this study, the 2.5 MV portal imaging is quantitatively characterized in terms of high‐ and low‐contrast resolutions and contrast‐to‐noise ratios using various phantoms. In addition, clinically acquired patient images of 2.5 MV are qualitatively compared with those of 6 MV and kilovoltage (kV) planar imaging. This study also provides guidelines for selecting imaging modes for different treatment sites.

## II. MATERIALS AND METHODS

The 2.5 MV and 6 MV portal and kV planar imaging modes have been evaluated quantitatively using various phantoms and qualitatively using images taken for the patient position verification. All images obtained in the phantom study were saved in DICOM format and processed in MATLAB (Mathworks, Natick, MA). A new imaging panel (aS1200) equipped with new TrueBeam linac has 43 cm×43 cm active area with 1280×1280 pixel resolution, terbium‐doped gadolinium‐oxysulphide (Gd2O2S: Tb) detectors, 25 frames per second, a dynamic range of 88 dB, and supports dose rates up to 7000 MU/min. On the front surface of the panel, a 1 mm conversion copper plate is used to attenuate low‐energy photons. The panel also has less backscatter than its predecessor due to the aluminum layer directly underneath the amorphous Silicon (aSi) array resulting in improved contrast. The pixel size is 336 μm. All imaging modes evaluated in this study were calibrated in the service mode before the experiments. The output of 2.5 MV and 6 MV beams was calibrated to deliver 1 cGy/MU at the depth of maximum dose. Whereas, the radiographic imaging dose from the Varian OBI is approximately 1−3 mGy per image, depending on imaging technique.^(24)^

### A. Leeds phantom

The high‐ and low‐contrast resolutions of the 2.5 MV portal imaging mode were evaluated using a Leeds phantom (TOR 18FG, Leeds Test Objects Ltd., North Yorkshire, UK) and compared to 6 MV portal and kV planar imaging mode. The Leeds phantom contains 21 line pair patterns for spatial resolution (0.5 lp/mm‐5.0 lp/mm) and 18 circular objects for low contrast detectability (0.9%‐16.7% contrasts). In this study, test objects for low‐contrast detectability and spatial resolution were used for evaluation. The Leeds phantom was placed on the Varian PerfectPitch 6‐DoF couch at 100 cm source‐to‐surface distance (SSD). The phantom was imaged with 2.5 MV and 6 MV portal imaging and kV planar imaging modes with the various thicknesses of solid water slabs placed on top of the phantom to simulate larger patients. The line pair patterns and circular objects on the phantom were used to quantify high‐contrast resolution (spatial resolution) and the low‐contrast resolution (low‐contrast detectability) of each imaging modes. In the OBI workspace, the highest line pair pattern resolved and the total number of object visible were recorded as the function of the solid water slab thickness. For better visibility of objects in the phantom, window and level were adjusted manually. For 2.5 MV and 6 MV portal imaging, a 30 cm×30 cm field size, using high‐resolution (Highres) imaging mode (1280×1280×16 resolution), and 3 MUs were used to create one image. For kV planar imaging, a field size of 30 cm×24 cm defined by Blade X and Blade Y was used.

The techniques for kV planar imaging were 50 kVp/75 kVp, 20mA/40mA, and 20 ms, with a large focal spot using a titanium filter. The higher kVp and mA were selected to compensate for the increased scatter and attenuation in solid water slabs. It should be noted that an even higher kV technique could have been selected to achieve higher contrast, given a certain thickness of solid water slab. However, in this study, a kV technique was chosen which did not saturate an image with no solid water slab added, which was selected to demonstrate the degradation of kV planar images for larger patient separation.

### B. RANDO male phantom

Contrast‐to‐noise ratios (CNRs) of bone (spine) to soft tissue were measured using an anthropomorphic RANDO male phantom (The Phantom Laboratory Inc., Salem, NY) in which lung, rib cage, and spine are constructed to mimic human organs in shape, effective atomic number, and mass density. The phantom consists of 2.5 cm slabs, allowing for film measurement and holes in grid configuration for dosimeter insertion. The dimension of the phantom is 24 cm in the anterior‐posterior (AP) and 34 cm in the lateral directions, measured at the level of Xiphoid process. CNRs of bone to soft tissue were calculated using Eq. (1) on lateral images adjacent to the spine where the soft tissue was relatively homogeneous. To simulate larger patients by increasing scatter and attenuation, solid water slabs were simultaneously added on both sides of the phantom to evaluate CNRs as a function of patient separation. For 2.5 MV and 6 MV portal imaging, high‐dose (3 MUs) and low‐dose (1.5 MUs) modes were selected (vendor preset). For kV planar imaging, thorax technique (100 kVp and 5 mAs, vendor preset) was selected. Image sizes for 2.5 and 6 MV imaging were 1280×1280 and 640×640 for 3 MUs and 1.5 MUs, respectively. Image size for kV was 1280×768.

(1) $$CNR=\frac{{\bar{Signal}}_{Bone}-{\bar{Signal}}_{Soft}}{\sigma_{soft}}$$

### C. BrainLab pelvis phantom

BrainLab pelvis phantom (Brainlab, Feldkirchen, Germany) was employed to obtain CNRs of the bone (spine) to soft tissue. The dimension of the phantom is 21 cm and 30 cm in AP and lateral directions, respectively. Similar to the methods in Materials & Methods section B above, lateral images were acquired using 2.5 and 6 MV portal and kV planar imaging modes with the addition of solid water slabs on both sides of the phantom. CNRs were calculated on all images acquired using all imaging modes using Eq. (1). The same imaging parameters as in the section above were used for MV imaging modes. Pelvis technique (140 kVp and 10 mAs, vendor preset) was selected for kV planar imaging.

### D. Patient images

For the purpose of position verification after patient positioning based on CBCT, an orthogonal pair of planar images was acquired using 2.5 and 6 MV portal and kV planar imaging modes. Images were obtained in AP and lateral directions for fractionated stereotactic radiosurgery (fSRS) for brain and head and neck and stereotactic body radiotherapy (SBRT) for lung and abdomen. For 2.5 and 6 MV portal imaging modes, 1.5 MUs and 3 MUs were delivered by vendor default to obtain a single image, respectively. The kV imaging technique was selected according to the treatment site, such as head, thorax, and pelvis. BrainLab, QFix H&N (Qfix, Avondale, PA), and Calypso (Calypso Medical Technologies, Inc., Seattle, WA) couch inserts compatible to the QFix 6DOF couch were used for SRS brain, head and neck, and other SBRT treatments, respectively. The two rails reinforcing a couch top were centrally positioned for all treatment and can be visualized in the AP images in SBRT treatments. Since the rails do not extend to the superior part of the couch top, rails are not visible in the AP images of SRS brain and head and neck. For visual confirmation after autoregistration, images were visualized using a content filter and compared with digitally reconstructed radiography (DRR). Content filter equalized signal intensities and sharpened edges of anatomical structures. Custom contrast window and level (CT Window [HU] from 100 to 1000 and clipping from ‐20 to 20) were used for DRR generation for better bony visualization.

## III. RESULTS

### A. Leeds phantom

**Figure 1:**
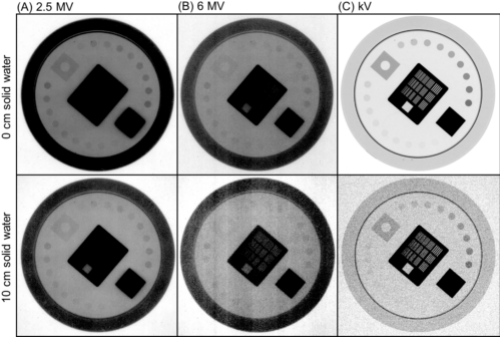
Low‐contrast detectability test for 2.5 and 6 MV portal and kV planar imaging modes using the Leeds phantom. The images in the first and the second row were taken with 0 cm and 10 cm solid water slabs placed to simulate increased side scatters and primary beam attenuation occurred in large patient. Window and level were adjusted heuristically to visualize all the contrast test disks. Field size for MV imaging: 15 cm (X)×15 cm (Y); for kV imaging: 30 cm (X)×24 cm (Y).

**Figure 2:**
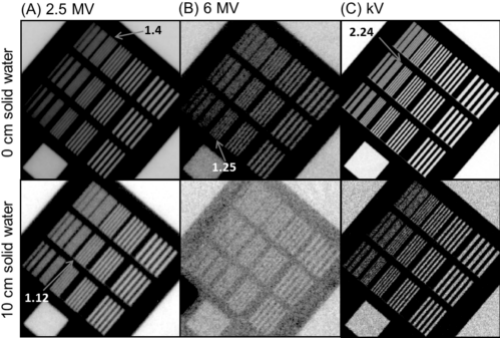
Spatial resolution evaluation using Leeds phantom. Central part of the images were cropped. Additional solid water slabs were used to mimic the situation of large patient in which side scatters and primary radiation beam attenuation are increased. Window and level were adjusted heuristically for optimal visualization of contrast object located peripherally. Field size for kV: 30 cm (X)×30 cm (Y; for MV: 15 cm (X)×15 cm (Y.

**Figure 3:**
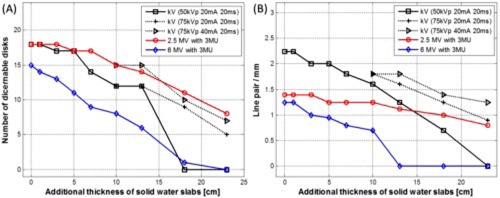
Low‐contrast detectability and spatial resolution with respect to additional thickness of solid water slabs.

Example images acquired for low‐contrast detectability and spatial resolution tests using all three imaging modes are presented in Figs. 1 and 2, respectively. To demonstrate image degradation due to increased scatter and attenuation of the primary radiation beam, images with 0 cm and 10 cm solid water slabs placed on the phantom are displayed in the first and second rows of the figures, respectively. The window and level was heuristically adjusted for the best visualization of low‐contrast test objects (disks in Fig. 1) and line pair patterns (Fig. 2). Imaging performance with 10 cm solid water slabs deteriorated significantly compared to 0 cm solid water slab due to increased side scatter and attenuation of the primary radiation beam caused by the additional solid water slabs. The quantitative analyses of both test results are plotted in Fig. 3 as the function of various thicknesses of solid water slabs. In the low‐contrast detectability test shown in Fig. 3(a), 18 disks were visualized using 2.5 MV portal and kV planar imaging modes with 0 cm solid water slabs, whereas only 15 disks were visualized using 6 MV portal imaging mode. As the beam path length increases, the low‐contrast detectability continually decreases. With 18 cm thickness of the solid water slabs placed on the phantom and 3 MUs, 2.5 and 6 MV portal imaging modes visualized 11 and 1 disks, respectively, while kV planar imaging mode imaged 0 disks with the kV technique: 50 kVp, 20 mA, and 20 ms. To increase penetration power, two new kV techniques with increased kVp and mA (75 kVp, 20 mA / 40 mA, 20 ms) were used, which improved the low‐contrast detectability resulting in visualization of 9 and 10 disks, respectively, at the same 18 cm thickness of solid water slabs.

With 23 cm solid water slabs, no disks could be visualized using 6 MV portal imaging, while 8 disks were visualized using 2.5 MV portal imaging.

In the spatial resolution test presented in Fig. 3(b), with 0 cm solid water slabs placed on the phantom, 2.5 and 6 MV portal imaging modes achieved 1.4 lp/mm and 1.25 lp/mm with 3 MUs, respectively, while kV planar imaging mode achieved 2.24 lp/mm with the kV imaging technique of 50 kVp, 20 mA, and 20 ms. With the additional 18 cm solid water slabs, the spatial resolution decreased to 1 lp/mm and 0 lp/mm for 2.5 and 6 MV portal imaging modes, respectively, while kV planar imaging mode achieved 0.71 lp/mm. Increased scatter and attenuation deteriorated the imaging power of 6 MV portal and kV planar imaging modes significantly and quickly in comparison to 2.5 MV portal imaging mode. With the new kV techniques, the spatial resolution increased to 1.25 lp/mm with 20 mA and 1.4 lp/mm with 40 mA, respectively, at 18 cm solid water slabs. With 13 cm solid water slabs, 6 MV portal imaging mode could not resolve the lowest line pair pattern (0.5 lp/mm), whereas 2.5 MV portal imaging mode could resolve 0.71 lp/mm with 23 cm solid water slabs.

### B. RANDO male phantom (thorax phantom)

**Figure 4:**
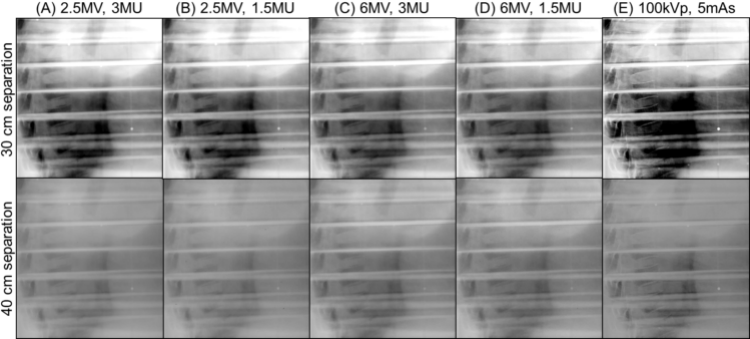
Lateral planar images of RANDO thorax phantom obtained from 2.5 and 6 MV portal and kV planar imaging modes. Solid water slabs were placed on both side of the phantom with equal thickness (in this example, 5 cm on each side). Field size for kV imaging: 30 cm×24 cm; for MV imaging: 40 cm×40 cm. Technique for kV planar imaging mode: 100 kVp and 5 mAs. 3 MUs and 1.5 MUs were delivered for both 2.5 and 6 MV portal imaging modes. Image resolution for kV: 1280×768, for MV with 3 MU: 1280×1280, and for MV with 1.5 MU: 640×640. Phantom dimension: 23 cm×30 cm in AP and lateral directions.

**Figure 5:**
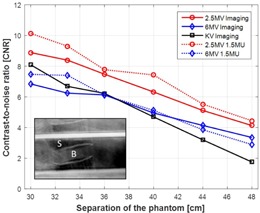
CNRs of bone to soft tissue with respect to the separation of the phantom. X‐axis indicates the separation of the phantom including the additional thickness of solid water slabs on the beam path. S=signal in the bone, B=background in the soft tissue. The phantom dimension: 23 cm×30 cm in AP and in lateral directions.

Using the anthropomorphic thorax phantom, the contrast‐to‐noise ratio of bone to soft tissue was evaluated as a function of thickness of solid water slabs placed on both side of the phantom. The solid water slabs were used to demonstrate the increased side scatter and attenuation of the primary radiation beam occurring in larger patients. Two sets of lateral images from each imaging modality with 0 cm and 10 cm thickness of water slabs are presented in Fig. 4. For MV imaging, 3 MUs and 1.5 MUs were delivered for single image acquisition. With 0 cm solid water slabs (lateral separation: 30 cm), major structures such as ribs, heart, spine, and lung were visualized in all imaging modes, as shown in the first row of Fig. 4. With 10 cm additional solid water slabs (lateral separation: 40 cm) shown in the second row of the figure, structures were less differentiable, and all images seemed to have the similar contrast. CNRs of bone to soft tissue with respect to the various thicknesses of solid water slabs (separation) are shown in Fig. 5. With 0 cm solid water slabs, 2.5 MV with 3 MUs and 1.5 MUs, 6 MV with 3 MUs and 1.5 MUs, and kV imaging modes, achieved CNRs of 8.9 and 10.1, 6.8 and 7.5, and 8.1, respectively. As slab thickness increases, CNRs of all imaging mode decrease with different degrees of deterioration. As an example, with 10 cm solid water slabs (40 cm separation), 2.5 MV with 3 MUs and 1.5 MUs, 6 MV with 3 MUs and 1.5 MUs, and kV imaging modes achieved CNRs of 6.3 and 7.4, 4.9 and 5.1, and 4.7, respectively.

### C. BrainLab pelvis phantom

**Figure 6:**
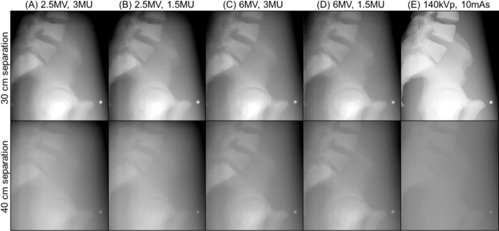
Lateral planar images of the pelvic phantom acquired using 2.5 and 6 MV portal and kV planar imaging modes. Solid water slabs were placed on both side of the phantom on the beam path with equal thickness (in this example, 5 cm on each side). Field size for kV imaging: 30 cm×24 cm; for MV imaging: 40 cm×40 cm. Technique for kV planar imaging: 140 kVp and 10 mAs. 3 MUs and 1.5 MUs were delivered for both MV portal imaging. Image resolution for kV: 1280×768, for MV with 3 MU: 1280×1280, and for MV with 1.5 MU: 640×640. The phantom dimension: 21 cm×30 cm in AP and lateral directions.

CNRs of bone to soft tissue as the function of various solid water slabs were evaluated in the BrainLab pelvis phantom in the same method as the thorax phantom. Figure 6 shows two sets of images acquired with 0 cm and 10 cm additional solid water slabs in the first and the second rows, respectively. With 0 cm solid water slabs (30 cm lateral separation), spine and pelvic bones were visualized in 2.5 and 6 MV portal imaging with 3 MUs and 1.5 MUs while kV planar imaging mode seemed to achieve higher contrast. With the addition of 10 cm solid water slabs (40 cm lateral separation), spine and pelvic bones were still well visualized with both MV portal imaging modes. However, the kV planar images taken with the same kV imaging technique were not visualized as much as 2.5 and 6 MV portal images. This is attributed to substantially higher phantom scatter and primary radiation beam attenuation from increased phantom thickness, which significantly degrades CNRs in kV imaging.

**Figure 7:**
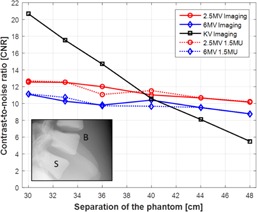
CNRs of bone to soft tissue in the pelvic phantom with respect to the lateral separation of the phantom. X‐axis indicates the separation of the phantom including the additional thickness of solid water slabs in the beam path. S=signal in the bone, B=background in the soft tissue. Pelvic dimension: 21 cm×30 cm in AP and lateral directions.

CNRs of bone to soft tissue in the BrainLab pelvis phantom were plotted with respect to additional solid water slabs in Fig. 7. With 0 cm solid water slabs (30 cm lateral separation), 2.5 MV with 3 MUs and 1.5 MUs, 6 MV with 3 MUs and 1.5 MUs, and kV imaging modes achieved CNRs of 12.6 and 12.7, 11.1 and 11.2, and 20.7, respectively. With an additional 10 cm of solid water slabs (40 cm lateral separation), the CNRs were reduced to 11.0 and 11.5, 10.4 and 9.7, and 10.6, respectively. With a separation greater than 40 cm, CNRs of kV is decreased compared to both portal imaging modes.

### D. Patient images

#### D.1 Fractionated stereotactic radiotherapy for brain

**Figure 8:**
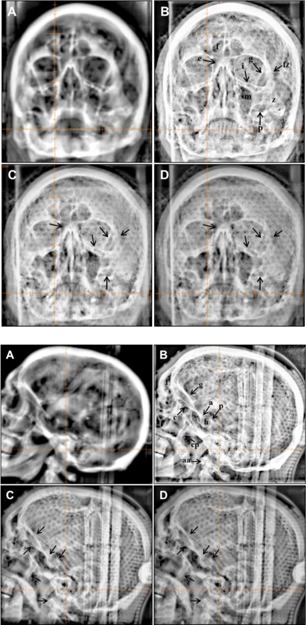
Posterior‐anterior (PA) images (top panels) used in fractionated brain SRS: (a) DRR, (b) kV (85 kVp and 5 mAs), (c) 2.5 MV portal image with 1.5 MUs, and (d) 6 MV portal image with 3 MUs. Left lateral images (bottom panels) used in fractionated brain SRS: (a) DRR, (b) kV (70 kVp and 5 mAs), (c) 2.5 MV portal image with 1.5 MUs, and (d) 6 MV portal image with 3 MUs. Cross hair indicates the treatment ISO. Content filter available on the Varian OBI workstation was applied for all images for visual verification after autoregistration f=frontal sinus, e=ethmoid sinus, i=inferior orbital rim, g=greater wing of sphenoid, m=maxillary sinus, fz=frontal process of zygomatic bone, z=zygomatic bone, p=petrous ridge, c=cribriform plate, s=sphenoid sinus, a=anterior clinoid processes, p=posterior clinoid processes (lateral image), h=hypophyseal fossa, cp=coronoid process of mandible, aa=anterior arch of the atlas

PA and lateral planar images obtained using all three imaging modes are presented in Figs. 8(a) and 8(b). In the PA images shown in Fig. 8(a), bony anatomy is better visualized with higher contrast and sharpness with 2.5 MV portal imaging mode compared to 6 MV portal imaging mode, but less than kV planar imaging mode. Some of anatomical landmarks indicated with arrows are frequently used for visual confirmation: f: frontal sinus, e: ethmoid sinus, i: inferior orbital rim, g: greater wing of sphenoid, m: maxillary sinus, fz: frontal process of zygomatic bone, z: zygomatic bone, p: petrous ridge. More anatomical structures were visualized in the lateral images as shown in Fig. 8(b). The portal image taken with 2.5 MV imaging showed higher contrast and sharpness than 6 MV portal image while kV planar imaging still kept the highest contrast with more details. Notable structures are labeled in the figures, f: frontal sinus, c: cribriform plate, g: greater wing of sphenoid, s: sphenoid sinus, a: anterior clinoid processes, p: posterior clinoid processes, h: hypophyseal fossa, cp: coronoid process of mandible, aa: anterior arch of the atlas.

#### D.2 Fractionated stereotactic radiotherapy for head and neck

**Figure 9:**
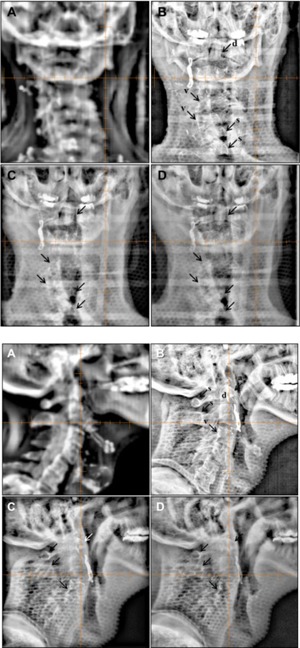
Posterior‐Anterior (PA) images (top panels) used for setup verification in the fractionated H&N SRS: (a) DRR, (b) kV (85 kVp and 5 mAs), (c) 2.5 MV with 1.5 MU, and (d) 6 MV with 3 MU. Right lateral images (bottom panels) used for setup verification in the fractionated H&N SRS: (a) DRR, (b) kV (70 kVp and 5 mAs), (c) 2.5 MV with 1.5 MU, and (d) 6 MV with 3 MU. Cross hair indicates the treatment ISO center. For visual confirmation of the setup after 2D‐3D autofusion, content filter was applied which was provided in the Varian OBI system. a=anterior arch of atlas, d=dens of axis, s=spinous processes, and v=vertebral body.

Notable bony structures in the head and neck images include the dens of axis (d) and vertebral bodies (v) which were imaged by 2.5 MV portal imaging mode with slightly higher contrast than 6 MV portal imaging mode (Fig. 9(a)). The spinal processes (s) were not visually different between both portal imaging modes. On the other hand, all bony structures were visualized with highest contrast and sharpness in kV planar imaging mode. In the lateral images, bony structures aforementioned were better identified than in the AP images. Anterior arch of atlas (s), dens of axis (d), spinal processes (s), and vertebral bodies (v) were visualized by 2.5 MV portal imaging mode with higher contrast than 6 MV portal imaging mode. kV planar imaging mode still maintained the highest contrast and resolution on lateral images (Fig. 9(b)).

#### D.3 Stereotactic body radiotherapy for lung

**Figure 10:**
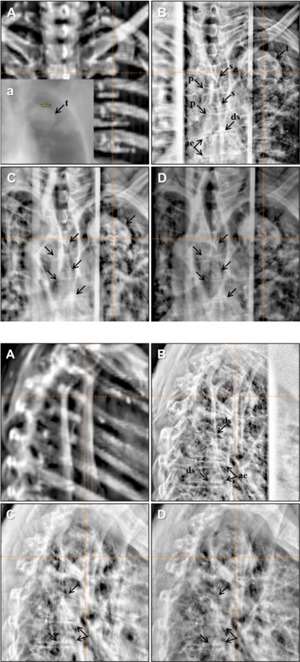
Anterior‐posterior (AP) images (top panels) used for setup verification in the SBRT lung treatment with the large bulky tumor: (a) DRR with insert showing DRR with different window, (b) kV (100 kVp and 5 mAs), (c) 2.5 MV with 1.5 MUs, and (d) 6 MV with 3 MUs. Right lateral images (bottom panels) used for setup verification in the SBRT lung treatment: (a) DRR, (b) kV (140 kVp and 10 mAs), (c) 2.5 MV with 1.5 MUs, and (d) 6 MV with 3 MUs. Cross hair indicates the treatment ISO center. For visual confirmation after autofusion, content filter was applied which was provided in the Varian OBI system. s=spinous processes, p=pedicle of vertebral arch, ds=intervertebral disk space, ae=upper and lower ambitus eminens. Patient separation through the ISO center: 19 cm in AP and 37 cm in lateral directions.

Verification images for lung SBRT from 2.5 and 6 MV portal imaging modes and kV planar imaging mode are presented in Fig. 10(a). In 2.5 MV portal images, spinous process (s), pedicle of vertebral body (p), and intervertebral disk space (ds) were imaged with higher contrast and sharpness with the half MU (1.5 MUs) than 6 MV portal images. The upper and lower ambitus eminens (ae) was visualized in only kV imaging clearly. Bulky lung tumors were visualized with highest contrast in 2.5 MV imaging among all three imaging modes. Additional DRRs are provided with the lung window leveling setting for better tumor visualization in the insert seen in Fig. 10(a). Ribs were visualized in kV planar image with highest contrast and resolution, which shadowed any underlying tumors. In the lateral images (Fig. 10(b)), 2.5 MV portal imaging mode was superior to 6 MV portal imaging mode in visualizing the intervertebral disc space (ds) and the upper and lower ambitus eminens (ae), although kV planar imaging mode was still superior. The upper and lower ambitus eminens was barely visible in all imaging modes, except one vertebral body in 2.5 MV portal and kV planar imaging modes. The tumor shown in the AP images was not visualized in the lateral images because of overlying high‐density structures such as ribs, humerus, and spine.

## IV. DISCUSSION

2.5 MV portal imaging mode available on the Varian Edge linear accelerator has been characterized in various phantoms for high‐ and low‐contrast resolutions in comparison to 6 MV portal and kV planar imaging modes. In addition, orthogonal pair of 2.5 MV portal images used for the patient position verification was compared to both 6 MV portal and kV planar imaging modes. 2.5 MV portal imaging mode has superior contrast compared to 6 MV portal imaging mode, although inferior to kV imaging. However, photons attenuate in the medium at different rates according to the photon energy. For example, after travelling 20 cm through water, 41% and 57% of the initial intensity of photons will be left for 2.5 and 6 MeV photons (maximum energy of 2.5 and 6 MV photon spectrum), respectively. On the other hand, only 3.9% of the initial intensity will remain for 120 keV photons (maximum energy of 120 kV photons).^(25)^ The different rates of attenuation were demonstrated in the phantom studies. With more solid water slabs in the beam path, the low‐contrast detectability, spatial resolution, and CNR declined remarkably in kV planar imaging mode with the same kV technique, although not in the 2.5 MV portal imaging mode. kV imaging can still achieve higher contrast with higher kV technique for a certain separation. Due to relatively less photoelectric interactions, the image quality of 6 MV portal imaging mode was not as good as 2.5 MV portal imaging mode even though 6 MV photons experience less attenuation than 2.5 MV. Tables 1 and 2 summarize the performance of each imaging modality in the Leeds phantom with 0 cm and 18 cm additional solid water slabs in the beam path and two anthropomorphic phantoms with 30 cm and 48 cm lateral phantom separation, respectively. (For CNR, lateral separation includes solid water slabs.) As shown in the tables, with additional 18 cm solid water and increased phantom separation, imaging performance of 2.5 MV portal imaging mode did not deteriorate as quickly as kV planar imaging mode, and is superior to 6 MV portal imaging mode in all imaging aspects evaluated. In the anthropomorphic pelvis phantom study (Fig. 7), CNRs with low dose (1.5 MUs) of both portal imaging modes were similar to the ones with high dose (3 MUs) because the low‐dose mode uses 2×2 binning (averaging) which increases signal and reduces noise. In the thorax phantom, similar trends were also found, as shown in Fig. 5. However, an unexpected deviation was presented. This was thought to be contributed by the artifacts from the multiple slices that make up the phantom.

**Table 1:** Changes in spatial resolution and low‐contrast detectability on Leeds phantom with additional solid water slabs

	*Spatial Resolution (lp/mm)*		*Low‐contrast Detectability*	
kV	2.24	0.80	18	0
2.5 MV	1.40	1.00	18	12
6 MV	1.25	0.00	5	0
Slabs	0 cm	18 cm	0 cm	18 cm

**Table 2:** Changes in CNRs on two anthropomorphic phantoms with additional solid water slabs. The lateral separation (Lat. Sep.) of the phantoms include the addition of solid water slabs shown in the table

	*CNR on Thorax Phantom*		*CNR on Pelvis Phantom*	
kV	10.7	1.0	9.0	1.5
2.5 MV	7.7	5.5	13.2	8.8
6 MV	6.8	5.1	11.0	8.0
Slabs	30 cm (Lat. Sep.)	48 cm (Lat. Sep.)	30 cm (Lat. Sep.)	48 cm (Lat. Sep.)

**Figure 11:**
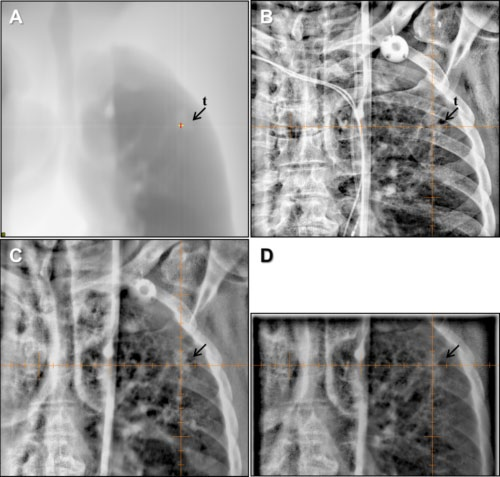
Posterior‐anterior image of lung SBRT: (a) DRR with soft tissue window to display the tumor location and shape (used only for this study), (b) kV (100 kVp, 5 mAs), (c) 2.5 MV with 1.5 MUs, and (d) 6 MV with 3 MUs. In (b), ribs overshadowed the underlying tumor resulting in poor tumor visualization.

From the comparison study of all three imaging modes shown in Figs. 8 to 10, 2.5 MV portal imaging mode is superior to 6 MV portal imaging mode in visualizing the bony anatomical structures and is comparable to kV planar imaging mode in the case of large patient separation. In lung SBRT, 2.5 MV also shows better performance in soft tissue imaging. As shown in Fig. 10, a bulky tumor was imaged with all imaging modes, but with different contrasts. In the specific case shown in Fig. 10, the ribs and the tumor were visualized with the similar contrast to each other. However, in another case shown in Fig. 11, the ribs visualized in the kV planar images overshadowed the underlying tumor whereas 2.5 and 6 MV portal imaging modes resulted in less of a shadowing effect caused by the overlying ribs.

Image quality of 2.5 MV portal imaging mode was compared with kV planar imaging mode as the function of patient separation in 21 abdominal and lung SBRT cases. In the comparison, 6 MV portal imaging mode was excluded in this comparison because this imaging mode was inferior to 2.5 MV portal imaging mode for all the images used in the study. The average patient separation for all patients in this study were 24.4±3.7 cm and 35.3±4.6 cm in AP and lateral directions, respectively (Table 3, lung patient) . Table 3 summarizes the statistical information of the patient separation when the lateral 2.5 MV images were comparable or inferior to kV images in the abdominal SBRT patients. The quality of lateral images of 2.5 MV portal imaging mode was comparable to kV planar imaging mode when the lateral separation of patients was greater than 38 cm (Table 3, abdomen patient, minimum lateral separation in the group). This is consistent with the pelvis phantom study. The quality of lateral images from 2.5 MV portal imaging mode was inferior to kV planar imaging mode when the lateral separation of patients was less than 36 cm (Table 3, abdomen patient, maximum lateral separation in the group). On the other hand, in the lung SBRT cases, 2.5 MV portal imaging mode was inferior to kV planar imaging mode for all the patients in the study (Table 3, lung patient,) even for patients with lateral separations greater than 44 cm, whose images for the same separation were comparable in the abdominal SBRT patients. This is because water‐equivalent path length in the lung (relative density: ~0.3) is shorter by about three times than in water‐like tissue (relative density: ~1).^(26)^

**Figure 12:**
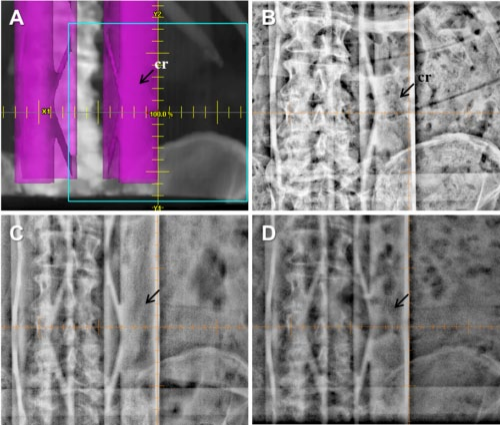
Couch rails imaged in the AP/PA images: (a) DRR with couch rail (cr) rendered with color in the Eclipse, (b) kV, (c) 2.5 MV with 1.5 MUs, and (c) 6 MV with 3 MUs.

In the authors' institution, couch rails are positioned in the center of the couch and radiation fields are configured not to enter the rails. This configuration of the rails and fields helps avoid any possible dosimetric uncertainty when the radiation beams is attenuated by the rails. Thus, in images, such as shown in Fig. 12, couch rails are imaged with patient anatomy. When the thicker portion of the couch rails overlap with anatomical landmarks, such as spinous process, upper and lower ambitus emines, and pedicles of vertebral body, visual confirmation of 2D‐3D autoregistration can be significantly affected. Couch rails can be also positioned away from center to the periphery of the couch. In this configuration, anatomical landmarks afore mentioned can be visualized well. However, radiation fields may need to be configured carefully in order to minimize dosimetric uncertainty caused by attenuation of the beam when they enter the rails.

**Table 3:** Imaging capability of 2.5 MV portal imaging mode as the function of patient separation in comparison with kV planar imaging mode. In the abdomen SBRT, 2.5 MV portal imaging mode was comparable to kV when the lateral separation was greater than 38 cm and 2.5 MV imaging mode was inferior to kV planar imaging mode when the lateral separation was smaller than 36 cm. 2.5 MV portal imaging mode was inferior to kV planar imaging mode for all lung SBRT patients. Avg=average, SD=standard deviation, Max=maximum, Min=minimum

	*Abdomen SBRT Patients*			
	*2.5 MV comparable to kV*		*2.5 MV inferior to kV*	
*Separation*	*AP*	*Lat*.	*AP*	*Lat*.
Avg	27.3	39.1	26.3	33.4
SD	1.9	1.3	3.5	2.7
Max	30.0	41.0	31.0	36.0
Min	26.0	38.0	22.0	30.0
	*Lung SBRT Patients*			
	*2.5 MV inferior to kV*		*Separation for all patients*	
*Separation*	*AP*	*Lat*.	*AP*	*Lat*.
Avg	22.4	35.0	24.4	35.3
SD	3.5	5.4	3.7	4.6
Max	27.0	44.0	31.0	44.0
Min	17.0	27.0	17.0	27.0
Unit: cm				

## V. CONCLUSIONS

A new portal imaging mode using 2.5 MV has been quantitatively characterized using various imaging phantoms and anthropomorphic thorax and pelvic phantoms. Spatial resolution and low‐contrast detectability of the 2.5 MV portal imaging mode was superior to conventional 6 MV portal imaging mode and did not degrade in the increased patient separations. Contrast‐to‐noise ratios of bone to soft tissue of 2.5 MV portal imaging mode were higher than 6 MV portal imaging mode and did not degrade with increased separations. During patient image analysis, 2.5 MV planar imaging mode preserved higher contrast and sharpness for bony structures with half the dose relative to 6 MV portal imaging mode. For larger separations, 2.5 MV portal imaging mode is an alternative imaging mode to kV planar imaging mode. 2.5 MV portal imaging mode would be a better option for cranial‐ and extracranial SRS than 6 MV portal imaging mode, which eventually assists in improving the accuracy of the image registration and beam delivery.

## ACKNOWLEDGMENTS

This work was supported in part by Varian Medical Systems, Palo Alto, CA.

## COPYRIGHT

This work is licensed under a Creative Commons Attribution 3.0 Unported License.

## Supporting information

Supplementary Material

Supplementary Material

Supplementary Material

Supplementary Material

Supplementary Material

## References

[1] Hurkmans C . Reply to Weiss et al. concerning our paper “Setup verification using portal imaging: review of current clinical practice”. Radiother Oncol. 2002;62(1):114–15. 10.1016/s0167-8140(00)00260-7 11166861

[2] Antonuk LE . Electronic portal imaging devices: a review and historical perspective of contemporary technologies and research. Phys Med Biol. 2002;47(6):R31–65. 11936185

[3] Marks JE , Haus AG , Sutton HG , Griem ML . The value of frequent treatment verification films in reducing localization error in the irradiation of complex fields. Cancer. 1976;37(6):2755–61. 949695 10.1002/1097-0142(197606)37:6<2755::aid-cncr2820370628>3.0.co;2-j

[4] Rosenthal SA , Galvin JM , Goldwein JW , Smith AR , Blitzer PH . Improved methods for determination of variability in patient positioning for radiation therapy using simulation and serial portal film measurements. Int J Radiat Oncol Biol Phys. 1992;23(3):621–25. 1612962 10.1016/0360-3016(92)90020-i

[5] Valicenti RK , Michalski JM , Bosch WR et al. Is weekly port filming adequate for verifying patient position in modern radiation therapy? Int J Radiat Oncol Biol Phys. 1994;30(2):431–38. 7928470 10.1016/0360-3016(94)90025-6

[6] Dawson LA and Jaffray DA . Advances in image‐guided radiation therapy. J Clin Oncol. 2007;25(8):938–46. 17350942 10.1200/JCO.2006.09.9515

[7] Kim J , Wen N , Jin JY et al. Clinical commissioning and use of the Novalis Tx linear accelerator for SRS and SBRT. J Appl Clin Med Phys. 2012;13(3):3729. 22584170 10.1120/jacmp.v13i3.3729 PMC5716565

[8] Kavanagh BD , McGarry RC , Timmerman RD . Extracranial radiosurgery (stereotactic body radiation therapy) for oligometastases. Sem Radiat Oncol. 2006;16(2):77–84. 10.1016/j.semradonc.2005.12.003 16564443

[9] Timmerman R , Papiez L , Suntharalingam M . Extracranial stereotactic radiation delivery: expansion of technology beyond the brain. Technol Cancer Res Treat. 2003;2(2):153–60. 12680797 10.1177/153303460300200212

[10] Timmerman RD , Forster KM , Chinsoo Cho L . Extracranial stereotactic radiation delivery. Sem Radiat Oncol. 2005;15(3):202–07. 10.1016/j.semradonc.2005.01.006 15983945

[11] Murphy MJ , Adler JR Jr , Bodduluri M et al. Image‐guided radiosurgery for the spine and pancreas. Comput Aided Surg. 2000;5(4):278–88. 11029160 10.1002/1097-0150(2000)5:4<278::AID-IGS6>3.0.CO;2-K

[12] Ryu SI , Chang SD , Kim DH et al. Image‐guided hypofractionated stereotactic radiosurgery to spinal lesions. Neurosurgery. 2001;49(4):838–46. 11564244 10.1097/00006123-200110000-00011

[13] Yin FF , Wang Z , Yoo S et al. Integration of cone‐beam CT in stereotactic body radiation therapy. Technol Cancer Res Treat. 2008;7(2):133–39. 18345702 10.1177/153303460800700206

[14] Herman MG , Abrams RA , Mayer RR . Clinical use of on‐line portal imaging for daily patient treatment verification. Int J Radiat Oncol Biol Phys. 1994;28(4):1017–23. 8138427 10.1016/0360-3016(94)90123-6

[15] Antonuk LE , Yorkston J , Huang W , Sandler H , Siewerdsen JH , el‐Mohri Y . Megavoltage imaging with a large‐area, flat‐panel, amorphous silicon imager. Int J Radiat Oncol Biol Phys. 1996;36(3):661–72. 8948351 10.1016/s0360-3016(96)00358-6

[16] Antonuk LE , el‐Nohri Y , Huang W . Initial performance evaluation of an indirect‐detection, active matrix flat‐panel imager (AMFPI) prototype for megavoltage imaging. Int J Radiat Oncol Biol Phys. 1998;42(2):437–54. 9788427 10.1016/s0360-3016(98)00210-7

[17] Kruse JJ , Herman MG , Hagness CR et al. Electronic and film portal images: a comparison of landmark visibility and review accuracy. Int J Radiat Oncol Biol Phys. 2002;54(2):584–91. 12243839 10.1016/s0360-3016(02)02955-3

[18] Bushberg JT . The AAPM/RSNA physics tutorial for residents. X‐ray interactions. Radiographics. 1998;18(2):457–68. 9536489 10.1148/radiographics.18.2.9536489

[19] Seibert JA and Boone JM . X‐ray imaging physics for nuclear medicine technologists. Part 2: X‐ray interactions and image formation. J Nucl Med Technol. 2005;33(1):3–18. 15731015

[20] Robberts DA , Hansen VN , Niven AC , Thompson MG , Seco J , Evans PM . A low Z linac and flat panel imager: comparison with the conventional imaging approach. Phys Med Biol. 2008;53(22):6305–19. 18936518 10.1088/0031-9155/53/22/003

[21] Orton EJ and Robar JL . Megavoltage image contrast with low‐atomic number target materials and amorphous silicon electronic portal imagers. Phys Med Biol. 2008;54(5):1275–89. 10.1088/0031-9155/54/5/012 19190362

[22] Parsons D , Robar J , Sawkey D . A Monte Carlo investigation of low‐Z target image quality generated in a linear accelerator using Varian's VirtualLinac. Med Phys. 2014;41(2):021719. 24506610 10.1118/1.4861818

[23] Connell T and Robar JL . Low‐Z target optimization for spatial resolution improvement in megavoltage imaging. Med Phys. 2010;37(1):124–31 20175473 10.1118/1.3267040

[24] Murphy MJ , Balter J , Balter S et al. The management of imaging dose during image‐guided radiotherapy: report of the AAPM Task Group 75. Med Phys. 2007;34(10):4041–63 17985650 10.1118/1.2775667

[25] Hubbell JH , and Seltzer SM . Tables of X‐ray mass attenuation coefficients and mass energy‐absorption coefficients from 1 keV to 20 MeV for elements Z = 1 to 92 and 48 additional substances of dosimetric interest. Retrieved May 19, 2015 from: http://www.nist.gov/pml/data/xraycoef/

[26] Van Dyk J , Keane TJ , Rider WD . Lung density as measured by computerized tomography: implications for radiotherapy. Int J Radiat Oncol Biol Phys. 1982;8(8):1363–72. 7141916 10.1016/0360-3016(82)90587-9

