# Supplementary figures and images for: Characterization and evaluation of 2.5 MV electronic portal imaging for accurate localization of intra‐ and extracranial stereotactic radiosurgery
Source: J Appl Clin Med Phys. 2016 Jul 8;17(4):268–84. doi: 10.1120/jacmp.v17i4.6247 (PMC5690040; doi:10.1120/jacmp.v17i4.6247)

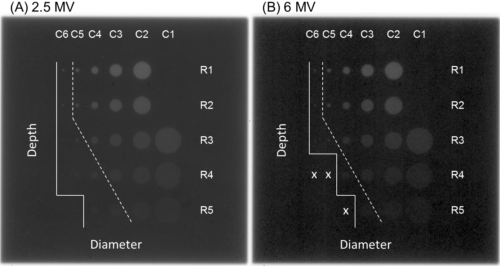

Supplement: Supplementary file 1 — Supplementary Material [file ACM2-17-268-s001.png]

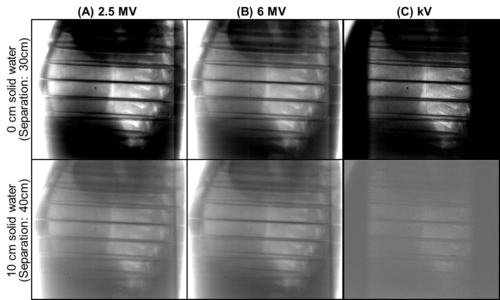

Supplement: Supplementary file 2 — Supplementary Material [file ACM2-17-268-s002.png]

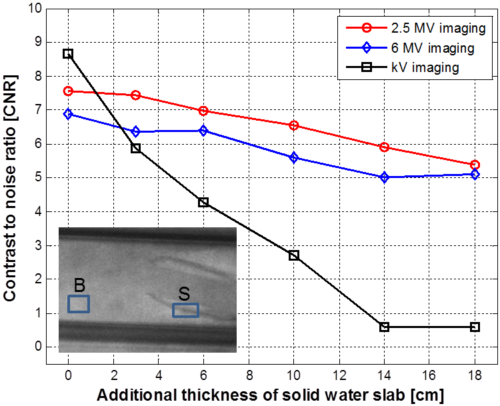

Supplement: Supplementary file 3 — Supplementary Material [file ACM2-17-268-s003.png]

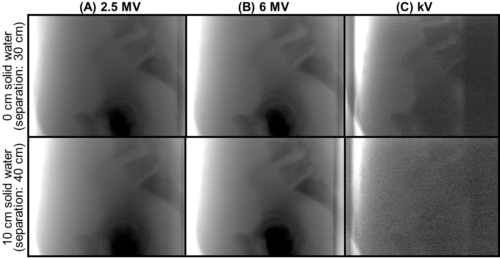

Supplement: Supplementary file 4 — Supplementary Material [file ACM2-17-268-s004.png]

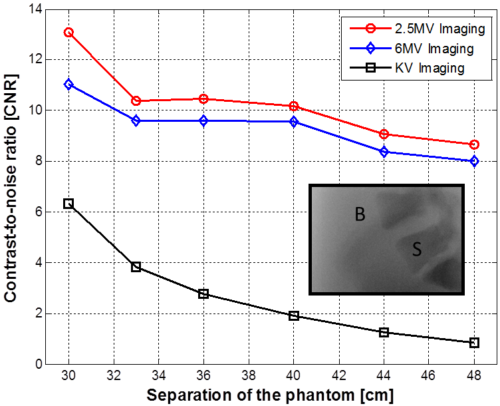

Supplement: Supplementary file 5 — Supplementary Material [file ACM2-17-268-s005.png]
